# Assessment of Total Antioxidant Capacity and Malondialdehyde Levels in Obese Patients With Periodontitis

**DOI:** 10.7759/cureus.75831

**Published:** 2024-12-16

**Authors:** Suzan M Taha, Faraedon M Zardawi, Chenar Anwar, Saeed A Mohammed, Hashim Mousa

**Affiliations:** 1 Department of Basic Sciences, University of Duhok, Duhok, IRQ; 2 Department of Periodontics, Qaiwan International University, Sulaimani, IRQ; 3 Department of Periodontics, Hawler Medical University, Erbil, IRQ; 4 Department of Periodontics, University of Duhok, Duhok, IRQ

**Keywords:** body mass index, malondialdehyde, obesity, oxidative stress, total antioxidant capacity

## Abstract

Background and objectives

Obesity is increasingly recognized as a serious chronic health issue worldwide. Numerous studies have highlighted its association with periodontal disease. Both obesity and periodontal disease may be connected through oxidative stress. The purpose of this study is to determine whether periodontal problems are linked to obese people’s greater vulnerability to oxidative stress.

Methods

A total of 120 individuals of both sexes (69 females and 51 males), with mean ages of 37.8 ± 9 years, were randomly assigned to one of three groups in a case-control study: 40 were in the control group, 40 were in the obese without periodontitis group, and 40 were in the obese with periodontitis group. The serum of the three groups was then evaluated for biochemical markers (total antioxidant capacity (TAC), malondialdehyde (MDA)) and a clinical periodontal parameter (clinical attachment level, CAL).

Results

The findings indicated that 30.7% of men and 41.6% of women participated in the study. Within each group, there was a significant difference (p < 0.001) in TAC and MDA levels between the obese and control groups, as well as between those with and without periodontitis. The obese group without periodontitis had a substantially larger mean waist circumference (93 ± 11.9) than the control group (86.0 ± 7.8), and the differences were statistically significant (p < 0.001).

Conclusion

Anthropometric and demographic measurements revealed no significant differences between obese individuals with periodontitis and those without it. Our results suggest that, in obese individuals, the onset of oxidative stress and reduced antioxidant capacity may act as a pathophysiological link to periodontitis.

## Introduction

Currently, the increasing prevalence of obesity has emerged as a major public health and economic issue worldwide. In 1997, the WHO classified obesity as a global epidemic. Today, approximately one-third of the worldwide population is affected by overweight or obesity [1].

Obesity is a significant public health challenge worldwide, impacting both developed countries with high living standards and developing nations with lower living standards [2]. Research has indicated that obesity and overweight are linked to some chronic illnesses, including atherosclerotic cardiovascular disease, diabetes mellitus, and periodontal disease [3, 4]. Inflammatory pathological conditions of the gingival and tooth-supporting tissues and periodontal diseases can cause partial or whole tooth loss. Clinical and radiological indicators of the breakdown of tooth-supporting tissues are known as periodontitis. Microbial dental biofilm is the primary cause of periodontal disorders [5, 6].

While subgingival plaque biofilm is the initial cause of periodontitis, tissue degradation appears to be caused by an aberrant host response to certain bacteria and their byproducts [7, 8].

Exaggerated inflammation, including the generation of reactive oxygen species (ROS) and proteolytic enzymes, is a hallmark of the aberrant response [9]. Systemic illnesses such as obesity, metabolic syndrome, diabetes, cardiovascular disease, and nonalcoholic fatty liver disease have been linked to periodontal disease in several studies [3, 10, 11]. Various methods have been used to identify oxidative stress, including assessing a reduction in total antioxidant capacity (TAC) or, more frequently, calculating the byproducts of oxidative damage to proteins, DNA, and lipids [12].

Oxidative stress is the hallmark of obesity, a chronic inflammatory illness [11]. The overproduction of reactive oxygen and nitrogen species by macrophages and monocytes in obesity is caused by decreased adiponectin levels and elevated levels of proinflammatory cytokines like tumor necrosis factor-alpha, interleukin-6, and interleukin-1beta, which raise oxidative stress [11].

The link between obesity and periodontal disease has gained notice recently and is currently a topic of discussion in dental and medical research. The body's tissue concentrations of antioxidants and ROS generation are delicate. Using clinical periodontal and oxidative stress indices as key endpoints [13], the study's null hypothesis is that obese individuals have higher levels of periodontal inflammation and local and/or systemic oxidative stress. Since there is no difference, the alternative hypothesis is not investigated.

Therefore, the aim of this study is to determine whether periodontal problems are linked to obese people's greater vulnerability to oxidative stress.

## Materials and methods

Patients groups

The research was conducted between September 2023 and March 2024 at the Duhok Central Laboratory and the Periodontics Department of the College of Dentistry in Duhok, Iraq. After obtaining approval from the Ethical Committee of the University of Duhok's College of Dentistry, the study's purpose was communicated to each participant, who then provided signed informed consent. A total of 120 volunteers participated in the study, comprising 69 women and 51 men, and were classified into three main groups. Group 1 served as the control group, consisting of 40 individuals with healthy periodontal tissue and no periodontitis (CAL = 0). Group 2 included 40 obese individuals with healthy periodontal tissue, while Group 3 consisted of 40 obese individuals with periodontitis. The individuals in both the obese (without periodontitis) and control groups were randomly selected from those referred to the college's periodontics department for periodontal therapy, provided they met the study criteria. Participants were required to be between the ages of 30 and 50, in good overall health, and must not have received any periodontal treatment in the six months leading up to the study. They also needed to refrain from taking any antimicrobial or anti-inflammatory medications in the three months prior, as well as being pregnant or nursing. Additionally, participants were not allowed to smoke, consume alcohol, or take any vitamin or antioxidant supplements during the six months before the study. For those with generalized moderate to severe periodontitis, inclusion criteria mandated that more than 30% of their sites must exhibit clinical attachment loss (CAL) of 3 mm or greater.

Clinical periodontal examinations 

To evaluate each participant's clinical periodontal health, measurements were taken from the free gingival margin to the base of the gingival sulcus for the control and obese groups, and from the cementoenamel junction to the base of the pocket for the obese with periodontitis group. A manual periodontal probe (PC-PUNC 15 Hu-Friedy, Chicago, USA) was utilized to measure six sites on each tooth. The CAL criteria included 3-4 mm for mild periodontitis and 5 mm or greater for severe periodontitis [14].

Blood samples, amounting to five milliliters, were collected from the antecubital veins between 8:00 and 9:30 a.m. The samples were centrifuged for 10 minutes at 3,000 rpm to obtain a clear supernatant, which was then stored in a refrigerator at -80 °C for future analysis of malondialdehyde (MDA) and total antioxidant capacity (TAC) levels. MDA levels were measured using a MDA assay that involves its reaction with thiobarbituric acid. This reaction results in the formation of colorful pigments, which facilitated the extraction of these colorful compounds in a distinct phase. The fluorescence intensity was measured at a stimulation wavelength of 520 nm and an emission wavelength of 550 nm, reported in nmol/mL [15].

TAC assay

To perform the TAC test kit technique (TAC assay kit ab65329 - abcam), 100 µL of Cu2+ of the working solution was added to each standard and sample well using the Colorimetric test Kit (www.abcam.com/products-kit). After that, the plate was combined and left on an orbital shaker, shielded from the light, to incubate for 90 minutes at room temperature. A microplate reader was used to measure the output at OD 570 nm.

Anthropometric parameters

BMI

The WHO has considered that obesity is an exposure variable [16]. An adult's weight in kilograms was divided by their height in meters squared to determine their body mass index or BMI. The study groups were classified as lean (normal weight (BMI 18.5-24.9 kg/m^2)^) and obese (BMI ≥30 kg/m^2^) based on the following criteria: underweight (BMI <18.5 kg/m^2^), normal weight (BMI = 18.5-24.9 kg/m^2^), overweight (BMI 25.0-29.9 kg/m^2^), and obese (BMI ≥30 kg/m^2^) [17].

Waist Circumference

After starting at the top of the hip bone, the tape measure was moved around the entire body until it was level with the belly button. Even in the rear, we checked to make sure it was straight and not too tight. The participants were told, however, to check the figure on the tape measure immediately after exhaling and not to hold their breath while measuring. Women's waist circumference must be greater than 88 cm (35 inches), and men's must be greater than 102 cm (40 inches) [17].

Statistical analysis 

The data were gathered and analyzed using the statistical software package SPSS 20. To assess the significance of correlations among the various variables, both Pearson and Spearman correlation analyses were performed. Descriptive statistics were represented through counts and percentages, while means and standard deviations were used for numerical data. A p-value of 0.05 or lower was considered statistically significant.

## Results

The results presented offer insights into the demographic and physiological traits of the studied population, encompassing gender distribution, age, anthropometric measurements (such as BMI and WC), biochemical parameters (TAC and MDA), and clinical parameters (CAL). Additionally, Table 1 details the variability around the means for each variable.

**Table 1:** Demographic data of the studied population. WC: Waist Circumference; TAC: Total Antioxidant Capacity; MDA: Malondialdehyde; CAL: Clinical Attachment Loss.

Variables	Mean ± SD
Sex Female no. (%) Male no. (%)	69 (41.6%) 51 (30.7%)
Age	37.8 ± 9
BMI (kg/m^2^)	28.2 ± 4
WC (cm)	90.4 ± 9.5
TAC (mM)	0.5 ± 0.4
MDA (ng/ml)	5.8 ± 2.5
CAL (mm)	1.0 ± 0.5

Table 2 illustrates that the gender distribution differences between the control and obese groups, as well as between individuals with and without periodontitis within each group, were statistically significant (p < 0.001). The mean BMI in the obese group was considerably higher (30.6 ± 3.1) than in the control group (23.6 ± 0.8), with these differences being statistically significant (p < 0.001). Additionally, within each group, obese individuals with periodontitis exhibited a higher BMI compared to those without, and this difference was also statistically significant (p < 0.001).

**Table 2:** Biochemical and anthropometric parameters of all groups. WC: Waist Circumference; TAC: Total Antioxidant Capacity; MDA: Malondialdehyde; CAL: Clinical Attachment Loss.

Variables	Control	Obese without periodontitis	P-value	Control	Obese with periodontitis	P-value
Sex Female no. (%) Male no. (%)	11 (27.5%) 29 (72.5%)	28 (70%) 12 (30%)	*˂0.000	11 (27.5%) 29 (72.5%)	30 (75%) 10 (25%)	*˂0.001
Age	32.3± 6.7	40± 8.3	*˂0.000	32.3 ± 6.7	40.9 ± 9.3	*˂0.001
BMI kg/m^2^	23.6 ± 0.8	30.6 ± 3.1	*˂0.000	23.6 ± 0.8	30.5±2.5	*˂0.001
WC cm	86.0 ± 7.8	93 ± 11.9	*˂0.000	86.0 ± 7.8	92.3 ± 6.5	*˂0.001
TAC mM	1.0 ± 0.4	0.3 ± 0.09	*˂0.000	1.0 ± 0.4	0.2 ± 0.07	*˂0.001
MDA ng/ml	2.7 ± 0.7	6.3 ± 0.6	*˂0.000	2.7 ± 0.7	8.4 ± 0.9	*˂0.001
CAL mm	0	0	0	0	3.1 ± 0.5	0

Table 2 further indicates a statistically significant difference between the control and obese groups regarding the mean serum levels of TAC and MDA, as well as between individuals with and without periodontitis within each group (p < 0.001).

The TAC levels were higher in the control group compared to the obese group, and individuals without periodontitis tended to have higher TAC levels than those with periodontitis. In contrast, MDA levels were elevated in the obese group relative to the control group, and those with periodontitis exhibited higher MDA levels compared to those without.

Our results also indicate that the average waist circumference was significantly greater in the obese group (93 ± 11.9) than in the control group (86.0 ± 7.8), with these differences being statistically significant (p < 0.001). Within each group, individuals with periodontitis exhibited significantly larger waist circumferences than those without the condition (p < 0.001).

The mean age in the control group was 32.3 years (standard deviation = 6.7 years), while in the obese group without periodontitis, the average age was 40 years (standard deviation = 8.3 years). Additionally, obese individuals with periodontitis were older than those without, and these differences were statistically significant (p < 0.001), as shown in Table 2.

Table 3 indicates that there were no significant differences in demographic and anthropometric measures between obese individuals with and without periodontitis. However, significant differences were observed in biomarkers of oxidative stress (MDA) and antioxidant capacity (TAC), as well as in clinical attachment loss levels, as illustrated in Table 3 below.

**Table 3:** Biochemical and anthropometric parameters of two groups. WC: Waist Circumference; TAC: Total Antioxidant Capacity; MDA: Malondialdehyde; CAL: Clinical Attachment Loss.

Variables	Obese without periodontitis	Obese with periodontitis	P-value
Sex Female no. % Male no. %	28 (70%) 12 (30%)	30 (75%) 10 (25%)	0.6
Age	40 ± 8.3	40.9 ± 9.3	0.6
BMI kg/m^2^	30.6 ± 3.1	30.5 ± 2.5	0.8
WC cm	93 ± 11.9	92.3 ± 6.5	0.7
TAC mM	0.3 ± 0.09	0.2 ± 0.07	*0.001
MDA ng/ml	6.3 ± 0.6	8.4 ± 0.9	*0.001
CAL mm	0	3.1±0.5	*0.001

## Discussion

This study marks the inaugural examination within Duhok City on the impact of obesity on TAC and oxidative stress (MDA) levels as well as their correlation with periodontal health in human subjects.

Our findings indicate a statistically significant difference between the obese group and the obese group with periodontitis regarding TAC and MDA levels.

The significant decrease in TAC levels observed in the obese group with periodontitis may be attributed to the chronic inflammatory processes associated with this condition. Such inflammation could result in oxidative damage to proteins, lipids, and DNA, ultimately contributing to the progressive destruction of the periodontal attachment apparatus. Additionally, other factors that may have contributed to the reduction in serum TAC levels include decreased uric acid levels, which serve as an important endogenous antioxidant. This reduction in uric acid may further exacerbate oxidative stress and impair the body’s ability to combat inflammation, leading to poorer periodontal health outcomes [18].

Both obesity and periodontal disease may collectively exacerbate inflammatory and oxidative conditions, leading to elevated local and systemic oxidative stress biomarkers [19]. According to Suresh's study [20], obese individuals with periodontitis exhibit higher levels of oxidative stress compared to obese individuals with a healthy periodontium.

Adipose tissue dysfunction can lead to systemic oxidative stress, which is linked to abnormal production of adipokines, contributing to various pathological systemic outcomes. Additionally, biomarkers indicating oxidative damage tend to be more sensitive in obese individuals and show a direct correlation with BMI and body fat percentage, as well as levels of low-density lipoprotein oxidation (LDL) and triglycerides (TG) [21]. In contrast, the levels of antioxidant defense markers are lower with body fat and central obesity [22, 23].

Shaimaa et al. [24] reported a strong association between leptin levels with lipid profile, BMI, and oxidative stress (MDA) levels, these connections contributing to obesity agree with our study.

Studies reported that increased production of reactive oxygen species and reduced antioxidant defense mechanisms may have been attributed to playing a role in both human and animal models of obesity [22, 25].

In the present study, both groups (obese with healthy periodontium, and obese with periodontitis) had a mean age of 40 years. Aging is intricately linked to systemic oxidative stress. Human aging involves two key aspects of oxidative stress: a decline in the availability of dietary antioxidants and an accumulation of oxidation byproducts within biological structures. Furthermore, aging cells and tissues are prone to compensate for the decreased dietary antioxidants by enhancing their production [26].

Furthermore, the current study found that females showed higher rates of obesity with periodontal disease than males. Gender appears to play a significant role in the mechanisms that trigger free radical production in professional athletes. Female athletes are more vulnerable to oxidative stress compared to their male counterparts. This variance in ferritin levels between genders may contribute to this disparity. Given the identified associations between oxidative stress and proteins involved in regulating iron transport and storage [27], further investigations are warranted to explore our findings thoroughly.

Limitations

The current study has some limitations, including a small sample size, which may have resulted in the underrepresentation of certain data. Additionally, the only oral clinical measurement employed to evaluate periodontal disease in all patients was the CAL. Non-surgical periodontal therapy (scaling and root planing) was not administered to obese patients with periodontitis, preventing the assessment of its clinical and biochemical effects through the measurement of oxidant and antioxidant defense markers before and after the treatment.

Authors recommend replicating this study including a larger sample size with the inclusion of an obese group with periodontitis that receives non-surgical periodontal therapy.

## Conclusions

Our findings suggest that obese individuals exhibit an increased susceptibility to the production of oxidative stress markers, evidenced by elevated levels of MDA and reduced levels of TAC. This change is associated with a higher risk of periodontal tissue destruction, as indicated by CAL in individuals with periodontitis. This highlights a significant connection between obesity and periodontal disease, as well as their impact on oxidative stress markers.
